# Decolonizing Bioethics in Africa

**DOI:** 10.20541/beonline.2016.0009

**Published:** 2016-11-22

**Authors:** Ademola Kazeem Fayemi, O.C. Macaulay-Adeyelure

**Affiliations:** 1 Department of Philosophy, University of Lagos, Akoka, Nigeria; 2 Department of Philosophy, Lagos State University Ojo, Nigeria

## Abstract

The global spread of bioethics from its North-American and European provenance to non-Western societies is currently raising some concerns. Part of the concern has to do with whether or not the exportation of bioethics in its full Western sense to developing non-Western states is an instance of ethical imperialism or bioethical neocolonialism. This paper attempts an exploration of this debate in the context of bioethics in sub-Saharan Africa. Rather than conceding that bioethics has a colonial agenda in Africa, this paper defends the position that the current bioethics trend in sub-Saharan Africa is an unintended imperialistic project. It argues that its colonizing character is not entirely a product of the Western programmed goals of training and institution building; rather, it is a structural consequence of many receptive African minds and institutions. Though bioethics in Africa is turning out as a colonizing project, one serious implication of such trend, if unchecked urgently, is that bioethics’ invaluable relevance to Africa is being incapacitated. This paper, therefore, attempts a decolonizing trajectory of bioethics in Africa. Contrary to the pretense of ‘African bioethics,’ which some African scholars are now defending, this paper through the logic of decolonization makes case for ‘bioethics in Africa’. In such logic, the principle of existential needs is prioritized over the principle of identity and authenticity that define African voice in bioethics.

## Introduction

The call for decolonization of disciplines in Africa is not new. Decolonization in this context means a process of self-critical awareness of foreseeing, discovering and avoiding hegemonic institutionalization as well as mental colonization of concepts and disciplines in contemporary African scholarship. Claude Ake^1^ led this intellectual vanguard in the Social Sciences; NgugiwaThiong’O^2^ and Chinweizu^3^ did same in politics of language and African literature; Okotp’Bitek^4^ is renowned for pioneering decolonization of Western religious concepts in African scholarship; Kwasi Wiredu^5^ is a prominent figure on the African philosophical scene championing the course of disciplinary decolonization; Hotep^6^ develops strategies for psychological decolonization of the colonized African minds; Lynda Smith^7^ came up with decolonizing the methodologies of research disciplines in Africa with emphasis on history. Decolonization is, therefore, well researched in the African context. The question is what next to decolonize: bioethics?

The case of bioethics is not likely to follow this well-thread path of decolonization of disciplines in Africa in a smooth fashion. Bioethics is a field and not limited by disciplinary boundaries. Bioethics is a normative and empirical consideration of moral issues and controversies about values emerging from biotechnologies and the health sciences. The global spread of bioethics as a field, which started developing over four and half decades ago in North-America and Western Europe, is unprecedented. In the spread of bioethics from its context of evolution to other parts of the world, the exportation has been wholesome involving the theories and principles, the practice and institutionalization. Unlike many other disciplines having root in the West, the exportation of bioethics, especially to Africa, is peculiar. Such peculiarity is not only in terms of its theories and principles, but also with particular reference to its institutionalization and professionalization as necessary concomitants. Its institutionalization has essentially been through initiatives of building research ethics capacity or provision of training and scholarships to Africans to study in mainstream bioethics (European and American) abroad. Whether this exportation is dubious, deserving suspect through decolonization or should be unwittingly welcomed as a positive development is a contentious matter.

Recently, Catherine Myser in an article “Reflecting on the Cultural Meanings and Social Functions of Bioethics” made remarks akin to the need for decolonization of bioethics in developing world, which Africa is euphemistically, in the front burner. In her words:

I believe the time is right in the development of the field for some “custom checks” on all sides of the national borders in question, exploring what is intentionally or unintentionally being developed and exported or imported, any unintended (negative) effects of exporting or importing “nonnative” species of bioethics into other countries, and relevant “local” developments or transformations that are taking place, whether noticed or unnoticed, outside or inside the countries in question.^8^

While the concern of Myser is not one strictly defined by motivation at a continental or regional level, her fervor for decolonization is clear in her call for a self-study, self-reflection, self-correction, self-re-awareness of the undue influences and negative unintended effects of exported bioethics to developing countries. Given the colonial and neo-colonial experiences of many states in Africa not only at the political space but also at the disciplinary level, it is apt to give Myser’s red flag about the ‘nonnative’ exportation of bioethics to developing states in Africa a further thought.

Few questions may help clarify and guide this new concern: is bioethics another instance of intellectual neo-colonization in Africa? Does the travel of bioethics to Africa in its present form have a colonial or neo-colonial underpinning be it explicitly or implicitly? In other words, to what extent is there ‘bioethical neocolonialism’^9^ in Africa? At what point does bioethics become an unwarranted exportation to Africa? What defines the need for decolonization of bioethics in Africa? What recipes are most fundamental to decolonizing bioethics in Africa? While there have not been studies that investigated these queries, this paper is an attempt to critically examine the issue of decolonizing bioethics focusing on the sub-Saharan African context.

## Is bioethics in Africa a colonial engagement?

In discussing whether bioethics in Africa is an imperialist agenda, which will in itself raise a normative question, it is important to understand if bioethics on its own regardless of its importation or exportation to Africa, is a residue of whiteness. The works by Christy Rentmeester and Catherine Myser are some of the few documented literatures on this supposition. Rentmeester thinks, for instance, that Western bioethics, with its origin in nineteenth-century physical and biological scientific and technological development in biomedicine, “sprang from the same objectifying tendencies that nourished nineteenth-century racism, sexism, and imperialism.”^10^ While Rentmeester’s claim can be argued to be mere association of bioethics evolution with the historical context and epochal events that marked the nineteenth-century, the blossom of imperialism during the period may not necessarily be connected with bioethics.

Questioning the American hegemony in bioethics, Myser asserts that “… unless researchers of diversity and difference *additionally* problematize white dominance and normativity and the white-other dualism, when they study and describe the beliefs and practices of other ethnic groups, their work merely legitimates and maintains “minoritized spaces” in bioethics.”^11^

The call on American (and Western) bioethicists to “‘decolonize’ their minds so that they will be more able to effectively ‘problematize,’ ‘displace,’ ‘decenter’ and ‘relocate’ ‘whiteness’ in their theory and practice presents formidable challenges for both white and nonwhite bioethicists.”^12^ While this claim on white dominance deserves some further investigation, the call for more critical alertness is more pertinent in the African context. Africa has always been victim of every form of Western hegemonic tendencies. Africa is an unconscious culprit of the globalization of Western bioethics. Many Western European and North American bioethics programs are designed in disseminating Western theories, practices and institutionalization of bioethics for ‘fix-it’ to trainees from developing world in the West.

It is on the foregoing account that De Vries Raymond and Leslie Rott put to question the goals of Western-centered bioethics education in the developed world. They likened the exportation of bioethics to developing world such as Africa to Christian missionaries which has greatly affected, whether for good or ill, African cultural mode of living. Just like the missionary gospel, “the gospel of bioethics is “good clinical practice,” “the Belmont report” and the “Declaration of Helsinki.”^13^ While missionaries care for the souls they minister to, bioethicists help create and write regulations in order to protect research subjects from harm and exploitation.

The striking contrast drawn by De Vries and Rott on the metaphor of missionary in relation to bioethics in developing world like Africa deserves some comments. Given the lesson of being dogged colonial imperialists, the West is now more suspect in the wholesome exportation of bioethics. Just as no longer would missionaries from the West be exported to other countries to evangelize, citizens from developing countries are brought to the West for training in order to contextualize the gospel in local culture. This is the strategy in bioethics just as in religion in post-colonial Africa. According to De Vries and Rott:

… those in the West who wish to bring the benefits of bioethics to the developing world have seen the value of indigenization … [as a solution to the exporting of] Western bioethics through training of [would-be] bioethicists in the United States (via the National Institutes of Health Forgaty International Center), Europe (via the Erasmus Mundus Masters programme in Bioethics, and the United Kingdom (via The Welcome Trust). Having learned the language and the logic of Western bioethics, trainees return to their home countries to spread the ‘gospel’.^14^

Contextualizing the above in the context of Africa, it might be appropriate to ask the question of the extent to which Western exportation of bioethics through training strategies is successful, and whether such attempts indeed constitute a mental colonization of the trainees as well as bioethical reflections in Africa. While this may require empirical study; in the absence of any conclusive quantitative survey, it is still worthwhile reflecting speculatively on the possible basic strengths of the argument.

If bioethics in Africa is now seen as a design, a product of enforcing carefully planned model of Western hegemony, as De Vroes and Rott intimated, should bioethics turns out to be imperialistic, it will be a fallacy of false cause to blame the Western countries that have sponsored Africans on bioethics programmes within their domain (the United States, the United Kingdom, and some European countries such as Belgium, Netherlands and Italy). The goal of the West in extending the benefits from their part of the world to the developing areas where such are yet to be experienced is not to be myopically interpreted as unwarranted domination. Though as a logical possibility, the tendency to think in this light is not unconnected with the historical antecedents of colonialism and many neo-imperialistic agendas the West is associated with.

While not holding brief unnecessarily for the West, the primary motivation of training Africans in the West and establishing collaborations in many parts of Africa through research institutions and projects is to share experiences and provide aid. Though secondary reasons may be inferred, the fundamental factor is sharing of experiences and expertise, which is unavoidable in a globalizing era. Some benefits, have no doubt, accrued directly and indirectly to Africans and African institutions through bioethics training programmes funded by some countries in the West. The training is an invaluable resource for members of ethics committees, biomedical researchers and other bioethics practitioners in different African states. Besides the direct impact of in building in the trainees, analytic and critical minds of being abreast with contemporary moral issues involved in biotechnology, the training programmes have indirect relevance for influencing and shaping public health and bio regulations in order to promote better health, life and care for the citizenry. On this note, bioethics education of Africans, whether in Africa or offshore, has a noble and laudable intent. It is, however, arguable that such “noble intent” notwithstanding, “it is not sufficient for bringing good results. An imbalance in power between would-be helpers and those to be helped creates a one-way flow of influence which not only diminishes the possibility of mutual enrichment, but also creates the possibility of unwitting harm.”^15^ In this state of affair, bioethics exportation to Africa is then an unintended imperialist project.

Perhaps, may be searching elsewhere for other sources besides bioethics education could be illuminating in understanding whether bioethics in Africa is imperialistic or not. To be sure, it is not meaningful discussing the colonization of bioethics in Africa without identifying the sources of bioethics colonization in the continent. Besides training of many African native bioethicists in the West, the fundamental sources of the mental colonization of bioethics in Africa includes: bio-law and regulations, institutionalization of bioethics, research collaboration and funding, and language.

In bio-law regulations in many African states, it could be argued that there is the dominance of “Western philosophical framework developed within institutional contexts of the West especially in relation to European and North American legal and policy frameworks.”^16^ For instance, the Kenyan biosafety law, which is an offshoot of the provisions of the Cartagena Protocol on biosafety, has some regulatory components that are premised on and indebted to principilism. The Kenyan Biosafety (Labeling) regulations seek to ensure that “information regarding genetically modified food, feed, or any other product is disseminated to the public so that consumers are able to make informed decisions.”^17^ In framing regulations as such, it is questionable if the West should be accountable for rarely censored policy framework on bio-regulations in many African states. The logic of ‘bate-passing’ of research ethics regulations in the West, especially as premised on principilism, with little or no modification in many regulations in African states may defy rational cogency.

Taking a look at the activities of bioethics bodies in Africa such as the Pan-African Bioethics Initiative (PABIN) and the West African Bioethics (WAB), one finds that many of their activities aimed towards aiding African states to participate in ethical debates, training and research at the international scene, are actually sponsored by some agencies in the West. Following the popular saying that “he who pays the piper dictates the tune,” it is suggestive that activities of such bioethics bodies are dictated by the funders. Exceptional cases to such lord-over rule are situations of collaborations between some of the institutional structures of bioethics in Africa and international bioethics bodies and institutes. West African Bioethics (WAB) training programme at the University of Ibadan, for instance, is collaborating with Global Health Reviewers (GHR), Training Resources in Research Ethics Evaluation (TRREE), as supported by the European and Developing Countries Clinical Trials Partnership (EDCTP).

Whether one calls it partnership, collaboration or sponsorship, the likelihood of hegemonic dominance is high because of power disparities. This is so because as long as there is little or no internal funding and impressive institution building by national governments in African states, such dominance is not unexpected. By no means does this relegate the importance of international collaborations; but such partnership may slip into unintended imperialism. For instance, it could be argued that the real intent of many of the collaborations with international institutions and projects such as the John Hopkins-Fogarty African Bioethics Training Program (FABTP) and the UNESCO’s “Assisting Bioethics Committees” (ABC) project is to indeed help African communities to build capacities in human subject research matters.

However, despite best intentions, as Myser critically and correctly notes, such efforts in bioethics in Africa “may be unwittingly advancing ideologies, power structures and institutions that in the end undermine the ethics enterprise and overall social justice.”^18^ How to decipher between intentional and unwitting imperialistic bioethics agenda in Africa remains an epistemic puzzle. Though instances of common experiences may intimate some inferences, such inferences are not conclusive. For instance, it is a common assumption among many Western researchers that Africa is the turning point for biomaterials especially with the paucity of strong and effective national and regional research regulatory bodies and ethical guidelines in some states in Africa.

Given the contextualization of bioethics training by few internationally supported bioethics institutions in Africa (e.g. WAB, FABTP), and the adaptation of the various international guidelines in line with subsisting regulatory frameworks in such states that they are based, the supposition that bioethics (at least in the sense of research ethics) in Africa is a colonial engagement may be difficult to substantiate without bias. Even if this is hypothetically the case, does it mean that decolonizing bioethics in Africa is an unnecessary exercise?

## Why decolonizing bioethics in Africa?

Decolonizing bioethics is about developing strategic mental, conceptual and structural resistance to the infiltration of real or foreseen hierarchies that tend (or may tend) to continue legacies of colonial hegemony whether implicitly or explicitly in every aspects of bioethics. Understood in this sense, decolonizing bioethics in Africa is therefore not a useless engagement. While the question of decolonizing bioethics in Africa has not been directly raised for reflective answer, the proclivity to it is discernible in the attempt by many bioethicists to help bring an end to what Garcia calls “the silencing, suppression, and exclusion of their vision and voices”^19^ in bioethics through what is now labeled ‘African bioethics.’ In other words, African bioethics has been popularly suggested as the best way to conceptually resist undue influences of Western bioethics hegemony in Africa.

Though without out-rightly labeling bioethics as a colonized field of engagement in Africa, Chikezie Onuoha provides some explanations relevant to the reason d’état for decolonizing bioethics in Africa. According to him, “the framework within which bioethics is done influences the result one gets …The theory and practice of bioethics have cultural underpinnings. Thus, culture is significant to the understanding an articulation of justifiable bioethics in a given society.”^20^ So for him, decolonizing bioethics will involve giving culture a pride of place in bioethics. This is pertinent because “there are divergent viewpoints and judgments from various cultures regarding many bioethical problems.”^21^ which have implications for how such problems are addressed. The possible conflation of ethnicity with culture is a grave danger glossed-over in the foregoing Onuoha’s explanations.

Cultural assumptions about health, illness, suffering, caring, life, personhood, community, death, and dying require that health care providers be sensitive and respectful of varied explanation models patients bring to the clinical encounter. Onuoha defends the need to ensure that bioethics not only in Africa but in a global context highlights moral or ethical pluralism. This is important because without the existence of alternative ethical frameworks that resonate with different cultural traditions and socio-political conditions, the commandeering of putative Western perspectives on bioethics is inevitable in the African context. Possible inference that can be drawn from the cultural assumptions about some issues of bioethics concern is that in engaging bioethics in Africa, linguistic consideration of African meanings of terms such as health, illness, personhood, dying and life are pertinent for the decolonization process in bioethics.

Onuoha’s supposition on the implicit need of decolonizing bioethics is in tandem with the view of Leigh Turner who argues that it is only when bioethics is culturally sensitive that it is authentic. To be authentic in this sense is to be free of external domination whether subtle or not. Turner notes that “Cultural explanations of health and illness, along with understandings of the appropriate social roles of family members and health care providers are interwoven with interpretations of what constitutes thoughtful moral conduct and moral reasoning.”^22^ In order to be respectful of the diverse explanatory models of healthcare receivers and givers in the clinical context, bioethics must be culturally sensitive. However, against this reason for the necessity of decolonizing bioethics, there are concerns that “such might promote cultural stereotypes giving rise to a trend that will probably over emphasize the value of particular communities, and deny variations in norms within specific groups.”^23^ Such an attempt “might fail to appreciate adequately commonalties across cultural communities.”^24^

For Maura A. Ryan, bioethical concerns are global, bioethical frameworks are not and the problem with Western bioethical thinking is that her frameworks, which are products of historical and cultural contexts are assumed to have timeless, universal validity. On this note, Ryan avers “…as bioethics has gradually developed a global consciousness, new voices from outside North America and Europe have emerged including from Africa, Asia, and Latin America, raising even more questions of adequacy and credibility.”^25^

Alerting on the need to be sensitive to uncritical acceptance of every sides of exportation of biotechnologies and its theoretical correlate, bioethics, to Africa, Godfrey Tangwa insists that “The globalization of Western technology should not be accompanied by the globalization of Western ways of thinking and acting, Western ways, manners and style of doing things, Western idiosyncrasies and eccentricities.”^26^

Tangwa, therefore, seeks an African bioethics, which also in the view of Kelvin Behrens^27^ applies indigenous African philosophy, thought and values to evolving bioethical issues and themes within African societies. In this same line of thinking is Clemetus Andoh who states that:

In order that African traditional ethical values are not seen as irrelevant for contemporary society and researchers, there is a serious need for bioethics in Africa to reclaim and return to the roots of African thinking so as to reconsolidate a true African authenticity. For bioethics to be authentically African, Africans must endeavor to root it, ground and fashion it according to their cultural norms as well as practical realities.^28^

For these scholars, the way to proceed on decolonization of bioethics in the African context is to pursue African bioethics. Defending a similar position, Munyaradzi Murove maintains that “the current discourse on bioethics in Africa is trapped in Western categories of thought.”^29^ Urging against avoidable bioethical colonialism in Africa, he makes the case for an authentic discourse on African bioethics which is cognizant of many Africans’ reliance on traditional medicine for their health care needs. Along this line, many of the literatures available on African bioethics focus mainly on the application of the cultural worldview to bioethical issues.

Each of the foregoing views is patterned on the principle of identity and authenticity. The logic of such principle is simply that to be real is to identify with one’s root and that what one used to be is what one ought to be. This seems to suggest that the provenance of a thing (in this case, bioethics) is what makes it either good or bad. This argument is a *non sequitor* as the Western origin of bioethics cannot be what makes it questionable. As an alternative, though, the advocates of the principle of identity and authenticity defend the necessity and veracity of African bioethics as the most plausible means of un-doing the colonizing subtleties and effects of bioethics on the African soil.

The supposition is that African bioethics will be an effective way of challenging uncritically assimilation of the dominant Western framework in bioethics analysis in Africa. The assumption of this model is that “ethical principles even when they are commonly accepted have to be applied and interpreted according to the perspectives of particular cultures and contexts”^30^ in order not to be paternalistic. A notable objection to this kind of reasoning can be found in Segun Gbadegin’s “Bioethics and Cultural Diversity.”^31^ For him, even if one were to accede to the claim that bioethics has its root in Western culture that does not mean that the problems addressed by the discipline are only of value to Western societies. For instance, cases of abuse of human subjects in experimental research is a common decimal everywhere. To the extent that the cases of research subjects’ abuses and the bioethical reflections and policies that evolved to stem them are universal, Gbadegesin will contend that the question of bioethics’ origin and efforts towards its decolonization in Africa is moot.

However, it needs to be critically noted, further, that the attempt to decolonize bioethics in Africa by opting for Africanizing bioethics exclusively in Africa has the danger of “dichotomizing different cultures as “radical others” to one another, promoting the tyranny of existing cultural practices, and obscuring the real ethical issues at stake.”^32^ Values that are advocated in bioethics such as respect for human dignity, human freedom and human care are not only universal in scope, they are also eternal verities. Thus, cultural differences can be seriously misconceived and misused in ways that some moral judgments and practical matters become easily entangled. The appeal to cultural differences as ‘colonial distance’ and serving as an ethical justification for rejecting those norms perceived as originating in the West and strongly advocated there is logically unsound. Such as attempt is a way of carpeting the moral difficulties by substituting statements about cultural practices for serious ethical examination. To quote Onuoha:

The cultural difference argument privileges cultural practices over ethical mandates; it implies, if not holds, that whatever is culturally authentic is automatically ethically defensible. This tyranny of culture over ethics can easily lead to moral relativism and even ethical nihilism.^33^

Further reasons can be given on why decolonizing bioethics in Africa through the channel of African bioethics is not cogent. African societies are no longer strictly in the traditional era, relying on traditional moral assumptions in African bioethical discourse and practices cannot, therefore, be sufficient. Nor can the content-variance of post-modern ethical traditions (such as we have in principlism, feminism, deontologism, utilitarianism, etc.) shaping bioethics in the Western world be exclusively taken as paradigmatic for encountering or doing bioethics in Africa. As the foremost Ghanaian philosopher, Kwasi Wiredu rightly noted:

Contemporary Africa is in the middle of a transition from a traditional to a modern society. This process of modernization entails changes not only in the physical environment but also in the mental outlook of our peoples, manifested both in their explicit beliefs and in their customs, and their ordinary daily habits and pursuits.^34^

The point to note from the above excerpt is that bioethics in contemporary Africa cannot be uniquely indigenous as many of the foregoing scholars have advocated, nor can it be entirely grounded on endogenous post-modernist ethical traditions.

## Decolonizing Bioethics in Africa: which way?

Though many bioethicists in Africa are aware of the need to decolonize bioethics only that the direction in which they beam their search light, which is a defense of African bioethics is questionable. To know whether that is the most plausible direction to path, few posers may help clear the doubts: what are Africans to decolonize in bioethics: the self-reflection of students and teachers of bioethics in Africa or the sensitivity of Western bioethicists or the impact its capable of bringing to bear on human wellbeing in the continent? Is the evolution of bioethics in Africa different from that of other disciplines which calls for decolonization in Africa had been made before now?

To address the first poser, it is not up to Africans to decolonize the minds of the Westerners, it is difficult to decolonize as well the minds of Africans who are (and will be) abroad for bioethics studies. Turning inside for indigenization of bioethics in what has been called African bioethics is not meaningful either. In addressing the second question above, decolonizing bioethics cannot take the same methodic, context and culture vagaries paradigm, which many of the decolonized disciplines in Africa tread.

If we consider bioethics as a forum of interdisciplinary commitment to the issues posed by the development of biotechnology, then decolonizing bioethics in Africa would be difficult if not impossible because it is not a discipline. It is a field; though currently, it is still tied within the ambit of academic African philosophers and few healthcare professionals in Africa. As it progresses into a field in the future, decolonization of bioethics as a field in Africa would have to be confronted with the challenge of whether to decolonize it as a field of professionals, or scholar inquiry, or policy making and activism, or consultants as research oversights, or biolaw expertise.

Just as decolonization itself necessarily involves continuous vigilance, conceptual self-awareness, critical self-questioning of goals, scope and strategies, in the African context, attention needs to be paid to the construction of bioethics that is contextually relevant and abreast of the processes of globalization. In other words, the self-conscious resistance enabled by decolonization temper would not be limited only to bioethics education in Africa. Its scope would extend to bioethics institutionalization, its politization, its concepts, and its goals within the broader prism of globalization. For the active beginning of bioethics decolonization in Africa, the bioethical consequences of globalization forces such as pandemics, terrorism, disasters, organ trade, medical-tourism, climate change, malnutrition, loss of biodiversity should take the front stage. Such decolonization will not discountenance global collaborations and recognition of exogenous values and cultures; it will engage them with close vigilance of questioning attitude. Focusing on the critical self-questioning goals of bioethics, based on the principle of priority, is the adequate and most plausible decolonizing trajectory to follow in Africa. The remaining part of this article shall justify this position.

## Toward a Decolonizing Agenda for Bioethics in Africa

While cultural, intellectual, political and technological backgrounds formed the emergence of bioethics in the Western world, such kinds of experiences in the West are unparalleled with that of Africa. In establishing African bioethics, the issue to consider must be what the historical, social, economic and political contexts of bioethics are which unavoidably [will] influence its nature, ambience and trajectory. Bioethics in Africa should be a critical engagement analysing “the social, political, and economic context of healthcare, research and science”^35^ in the emerging global order with its confluence of values.

The reality in many African societies today, particularly in the sub-Saharan part, seems to be that the impacts of globalization present new bioethical challenges. Examples of issues having a global nature with serious bioethical underpinning in the African world and elsewhere include: “pandemics, organ trafficking, climate change, hunger, malnutrition and obesity, corruption, bioterrorism, disasters and humanitarian relief, bio-piracy and loss of diversity, and degradation of the biosphere.”^36^ Given the social, health and moral challenges, which are the bane of human flourishing and dignity in Africa, bioethics in Africa needs be more critical of the neo-liberalist values concomitant to globalization, and consequently worsening the African social and humane condition. Behrens thoughtfully captures the undermining of human well-being in sub-Saharan African healthcare:

The inability of hospitals to provide patients with medication, the failure of poorly serviced equipment, and the non-payment of service providers reflect a health system in crisis. Maladministratio n and incompetence place countless patients at risk. Accounts of practitioners abusing state resources for personal gain at the expense of patients, private health sector fraud and over-servicing, and increasing numbers of professionals falling foul of the ethical standards of the Health Professions, [medical tourism, health inequity, exploitation of participants in medical research, unimaginable risks in biological samples and banking] … point to an ethical crisis [in the health sector with adverse effects on not only human dignity but also other spheres of human existence in Africa].^37^

Though biotechnologies create some moral problems that are conventionally of interests to bioethicists, the African prevalent healthcare problems and experiences seem to suggest more salient moral problems occasioned, in part, by the neo-liberal market ideology undergrounding the healthcare system. Bioethicists in Africa are not doing enough to expose, analyze, and criticize how the confluence and domineering of values reinforced by neoliberalism have continued to have pernicious effects on the well-being of the Africans. Rather than espousing how values such as cooperation, solidarity, cooperation, interconnectedness of human beings and other biotic components can be instrumental in meeting the yearnings and aspirations for improved human wellbeing and dignity of the Africans, many African scholars have taken the path of defending African bioethics as a means of decolonizing bioethics in Africa. While research ethics is gaining more prominence in Africa, for the most part, a decolonizing agenda of bioethics in Africa cannot afford to attenuate the frontiers of health care ethics.

Without unnecessary dichotomization of the thin lines between bioethics and healthcare ethics, bioethics is a broad area of enquiry covering all ethical issues in medicine, the life sciences and the environment.^38^ However, healthcare ethics is an interdisciplinary field that investigates moral problems in clinical, organizational, professional, and research issues related to human health.^39^ Since research ethics is embedded in healthcare ethics, and healthcare ethics is subsumed under bioethics, a decolonization agenda for ‘bioethics in Africa’ would be a label for understanding: (i) how bioethicists working in Africa (be it Africans or non-Africans) respond to the effects and impacts of the processes of globalization on issues of bioethics relevance; (ii) an evaluation of the bioethical challenges and problems in the continent with reference to using African ethical ideas and principles in relation to such concerns; (iii) how values and ideas from the other parts of the world harmoniously integrate (or can be domesticated) in providing moral directions in specific and concrete bioethical topic in Africa.

Though many of today’s biomedical ethical concerns such as genomics, genetic manipulation and third generation sequencing, sex selection, transhumanism and human enhancement, nanotechnology, cloning and new reproductive technologies, are global in nature, their impacts in sub Saharan African societies are marginally low. This point is relevant when it is compared to healthcare ethics concerns in the emerging global order: pandemics, organ trade, participants’ exploitation in multicenter-clinical trials, injustice in access to healthcare, poor resource allocation to health sector made worsen with its mismanagement, palliative care and end-of-life issues, bioterrorism, bio-piracy, genetic modified organisms (GMOs) to name a few.

Making a case for ‘bioethics in Africa’ rather than ‘African bioethics in Africa’ as the goal of decolonizing bioethics in Africa does not mean that studying the latter is theoretically irrelevant. It only implies that it is isolated from the more fundamental existential reality of the present African condition. While Africa cannot be indifferent to the multifold of problems generated by the globalization of biotechnologies, the low state of biotechnology in many parts of the continent and the dehumanizing impacts of globalization signal the direction to intensive the decolonization of bioethics in the continent. Choosing bioethics in Africa as the next field of African studies to decolonize aligns with the principle of priority which is prima facie in nature. This is because many of the moral problems emerging in the process of globalization in African healthcare system have direct impacts on the average Africans. Bioethics in Africa could be of immediate focus while pure African bioethics may progressively be developed as its necessity evolves. Azetsop is, therefore, correct in his remarks that “Bioethics has had significant effects in the health domain in industrialized countries, but not yet in African countries where it has yet to impact healthcare delivery and health policy.”^40^

Rather than dispensing intellectual efforts on a unique African bioethics, developing the genre of ‘bioethics in Africa’ is the most cogent way of decolonizing bioethics in the sub-Sahara. This is because it has indisputable advantages over the identity reference and reverences which ‘African bioethics’ is laden with.^41^ ‘Bioethics in Africa’ allows the opportunity of censoring the domineering values in globalization; it offers the theoretical platform for harnessing critically, the scope and circumstances of cultural, moral and aesthetic value-laden ideas to be accepted from any other parts of the world (be it oriental or occidental). This is without discountenancing the salient aspects of traditional African ethical values that are still relevant in the specific context of healthcare dilemmas in contemporary Africa. ‘Bioethics in Africa’, as a product of and response to moral pluralism, will allow for interdisciplinary collaborations (as opposed to being tied to the disciplinary apron string of indigenous African philosophical ethics). It will also ensure peaceable cross-cultural collaborations and respect for values of non-African natives inhabiting or receiving healthcare in Africa.

‘Bioethics in Africa’ is an application of bioethical ideas and moral commitments irrespective of their provenance in the consideration of moral problems in emerging from the effects of globalization on healthcare and human wellbeing in Africa. Moral ideas, theories and principles, whether from Africa, West, or the East are prima facie applicable in so far as they are proportionately and significantly integrative in the resolution of a specific morally problematic situation. One of the attractions of ‘bioethics in Africa’ is that it adds to the diversity of bioethical visions and horizons in contemporary times. Most importantly, the tasks for ‘bioethics in Africa’ are worth courting with. For one, it places a moral duty on bioethicists in Africa to ensure that their intellectual preoccupations have impacting bearings on human well-being as constrained by globalization values and local healthcare challenges. For the other, it propagates a commitment to the construction of values that contribute to the growing international dialogues in many areas of global bioethics. Such phrase as ‘bioethicist working in Africa’ should be more encouraged and popularized to ‘African bioethicist’ because of the former geographical neutrality. While reference to ‘African bioethicists’ is not fundamentally problematic as it could be a geographical marker of point of origin, the thin line between this mere geographical location and Africanness of content idea of the bioethics field makes it not worth courting with.

## Conclusion

From the foregoing, this article establishes that decolonizing bioethics in Africa is a necessity. ‘Bioethics for Africa’ is bioethics colonized and imperialized. As a self-conscious process, decolonizing bioethics in Africa requires sensitivity to goals which bioethics can bring about in Africa and not the degree to which it promotes African identity in the realm of bioethics. For this reason, ‘African bioethics’ is not the path to navigate for a sustainable decolonization of bioethics in Africa. As an ongoing process, decolonization requires persistent vigilance and self-questioning on bioethics issues and problems as they relate to contemporary African experiences.

Decolonizing bioethics on the African soil is an urgent imperative in order to avoid the supremacy of ‘Africaness identity’ vestige of some African bioethicists in having structural edge on the institutions of bioethics in Africa. To avoid the unwittingly imperialistic consequences of Western or Asian interventions and collaborations in bioethics education in Africa, there is need for robust funding of bioethics programmes and building of institutions at different levels by African governments and stakeholders. As decolonization process involves sound methodologies, there is need for a robust methodological inquiry in bioethics in Africa. This is a task for future studies in order to make the field of bioethics in Africa vibrant.
